# Rapid Typing of Extended‐Spectrum β‐Lactamase (ESBL)‐ and Metallo‐β‐Lactamase (MBL)‐Producing Enterobacterales Using Fourier Transform Infrared (FT‐IR) Spectroscopy

**DOI:** 10.1002/mbo3.70218

**Published:** 2026-02-02

**Authors:** Yasuhide Kawamoto, Kosuke Kosai, Mika Murata, Kenji Ota, Fujiko Mitsumoto‐Kaseida, Norihito Kaku, Hiroo Hasegawa, Koichi Izumikawa, Hiroshi Mukae, Katsunori Yanagihara

**Affiliations:** ^1^ Department of Laboratory Medicine Nagasaki University Hospital Nagasaki Nagasaki Japan; ^2^ Department of Laboratory Medicine Nagasaki University Graduate School of Biomedical Sciences Nagasaki Nagasaki Japan; ^3^ Department of Infectious Diseases Nagasaki University Graduate School of Biomedical Sciences Nagasaki Nagasaki Japan; ^4^ Department of Respiratory Medicine Nagasaki University Graduate School of Biomedical Sciences Nagasaki Nagasaki Japan

**Keywords:** antimicrobial resistance, molecular typing, outbreak, phenotypic similarity

## Abstract

This study investigated the usefulness of the IR Biotyper, which types bacterial strains using Fourier transform infrared (FT‐IR) spectroscopy, against extended‐spectrum β‐lactamase (ESBL)‐ and metallo‐β‐lactamase (MBL)‐producing Enterobacterales. Sixty‐six clinical isolates (20 ESBL‐producing *Klebsiella pneumoniae*, 15 IMP‐producing *K. pneumoniae*, and 31 IMP‐producing *Enterobacter cloacae* complex isolates) were analyzed using the IR Biotyper, pulsed‐field gel electrophoresis (PFGE), multilocus sequence typing (MLST), and whole‐genome single‐nucleotide polymorphism (wgSNP) analyses and the results were compared. Of the 20 ESBL‐producing *K. pneumoniae* strains analyzed, the IR Biotyper detected three clusters. Of these clusters, two were determined as respective clusters by PFGE and wgSNP analyses, and the strains included in each cluster showed the same STs. The IR Biotyper detected three clusters in the analysis of 15 IMP‐producing *K. pneumoniae* strains. Of these clusters, strains in the two clusters showed high concordance with PFGE, MLST, and wgSNP analyses. The IR Biotyper identified six clusters among the IMP‐producing *E. cloacae* complex isolates. These results were fully concordant with those of PFGE, MLST, and wgSNP analyses in the two clusters. The range of adjusted Rand index was 0.734–0.967 between the IR Biotyper and PFGE and 0.658–0.857 between the IR Biotyper and MLST or wgSNP analyses. This study demonstrated the performance of IR Biotyper for the detection of clonal similarities in ESBL‐ and IMP‐producing Enterobacterales and it might be useful for outbreak investigation.

## Graphical abstract

Clinical isolates of *Klebsiella pneumoniae* and *Enterobacter cloacae* complex were analyzed using FT‐IR spectroscopy and genotyping methods. High concordance was observed, demonstrating the potential of FT‐IR spectroscopy as a rapid screening tool. Some discordant results highlight the need for confirmatory genotyping in outbreak investigations.

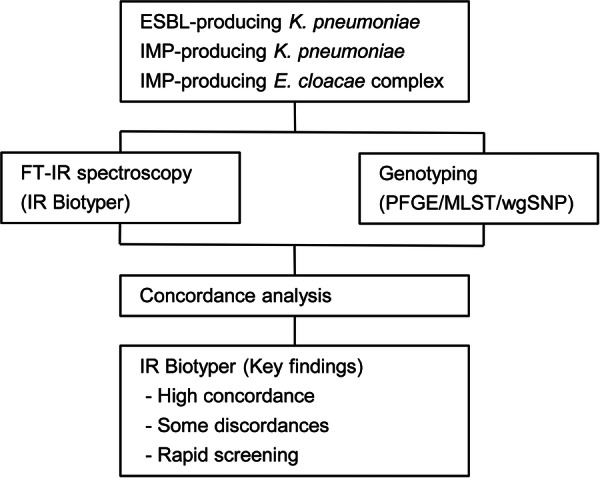

## 1 Introduction

The spread of carbapenemase‐producing Enterobacterales (CPE) and extended‐spectrum β‐lactamase (ESBL) producers is a major global concern. Several CPE outbreaks have been reported, and infections due to the bacteria contribute to unfavorable patient outcomes in some cases (Fukigai et al. 2007; Ducomble et al. 2015; Budhram et al. 2020). When outbreaks of drug‐resistant bacteria are identified via routine surveillance for antimicrobial susceptibility, genotyping using standard methods, such as pulsed‐field gel electrophoresis (PFGE), conventional multilocus sequence typing (MLST), and whole genome sequencing (WGS)‐based typing are usually required to confirm bacterial transmission among patients. However, these genotyping methods are time‐consuming and labor‐intensive.

Fourier transform infrared (FT‐IR) spectroscopy generates spectra by quantifying the absorption of infrared light of cellular components, such as carbohydrates, lipopolysaccharides, nucleic acids, proteins, and lipids. The FT‐IR spectrum reflects the overall cell composition and can be a distinctive fingerprint of each sample (Martak et al. 2019; Hu et al. 2021). The IR Biotyper system (Bruker Daltonics GmbH & Co. KG) is an automated typing system for bacteria using this technology, and the system uses the spectra of carbohydrates for bacterial typing (Martak et al. 2019). The procedure is simple and requires no more than 3 h per assay.

In this study, we analyzed the performance of the IR Biotyper for typing CPE and ESBL producers, compared to comparator methods, and discussed the usefulness of the method in clinical settings.

## 2 Methods

### 2.1 Study Design and Bacterial Isolates

We analyzed 66 clinical isolates (20 ESBL‐producing *Klebsiella pneumoniae* isolates and 46 metallo‐β‐lactamase [MBL]‐producing Enterobacterales [15 *K. pneumoniae* and 31 *Enterobacter cloacae* complex isolates]), which were isolated from Nagasaki University Hospital between January 2009 and January 2016. The strains used for comparisons were isolated from different patients. The characteristics of these isolates have been reported in our previous studies, and all MBLs were of the IMP‐type (Higashino et al. 2017; Kawamoto et al. 2018, 2019; Yamakawa et al. 2019). NCTC 13439 and BAA‐2146 were used as reference strains for *K. pneumoniae*.

### 2.2 FT‐IR Spectroscopy

Bacteria were cultured at 37°C for 24 h on trypticase soy agar (Becton Dickinson). The assay was performed using an IR Biotyper kit (Bruker Daltonics GmbH & Co. KG), according to the manufacturer's instructions. Bacterial colonies were collected using a 1‐μL loop, suspended in 50 μL of 70% ethanol (Wako Pure Chemical Industries Ltd.) in a Bruker suspension vial containing inert metal cylinders, and then mixed using a vortex mixer for 1 min. Subsequently, 50 μL of deionized water was added to the sample and mixed; then, 15 μL of the suspension was applied to at least four spots on the silicon microtiter plate and dried for 30 min at 37°C. The dried plate was assayed using the IR Biotyper (Bruker Daltonics GmbH & Co. KG) and analyzed using IR Biotyper software v2.1 (Bruker Daltonics GmbH & Co. KG). A dendrogram was generated based on the results of hierarchical cluster analysis (HCA) with the exploration method, Euclidean average mean spectra. The data of each strain in the dendrograms are expressed as the average of the results obtained from a single assay performed in quadruplicate. The IR Biotyper system automatically provided cut‐off values based on the results of the average linkage using Euclidean distance, and the values were adjusted in consideration of the results of comparator methods. The same isolates were assayed at least twice using the IR Biotyper; furthermore, we assessed whether the same isolates were classified into the same clusters between separate assays using the IR Biotyper. If the same isolates were classified into different clusters between the first and second assays using the IR Biotyper, an additional assay was performed for those strains, and the result was considered the confirmed result when it showed concordant clustering with either the first or second result.

### 2.3 Comparator Methods

PFGE, conventional MLST, and whole‐genome single‐nucleotide polymorphism (wgSNP) analyses were used as comparator methods to assess the results obtained using the IR Biotyper system.

Some sequence types (STs) of ESBL producers determined by the conventional MLST and all STs and PFGE results of IMP producers in *K. pneumoniae* have been reported previously (Higashino et al. 2017; Yamakawa et al. 2019). The STs of the remaining ESBL‐producing *K. pneumoniae*, IMP‐producing *E. cloacae* complex, and reference strains were determined, as described previously, with a few modifications (Yamakawa et al. 2019; Diancourt et al. 2005; Miyoshi‐Akiyama et al. 2013). Allele sequences and STs were determined according to *K. pneumoniae* MLST (http://bigsdb.pasteur.fr/klebsiella/klebsiella.html) and *E. cloacae* MLST databases (https://pubmlst.org/organisms/enterobacter‐cloacae).

PFGE for ESBL‐producing *K. pneumoniae* and IMP‐producing *E. cloacae* complex was performed by Miroku Medical Laboratory Inc., according to the manufacturer's instructions and as described in the textbook (Japanese Association of Medical Technologists 2003), with a few modifications. Briefly, *SpeI* was used as the restriction enzyme and electrophoresis was conducted at 6 V/cm with a field angle of 120°, using three sequential blocks with switch times ramped from 5.3 to 32.3 s for 4.9 h, from 32.3 to 44.5 s for 7.7 h, and from 44.5 to 49.9 s for 7.1 h, for a total run time of 19.7 h. A Dice coefficient‐based dendrogram was generated according to the unweighted‐pair group method with arithmetic mean (UPGMA) using BioNumerics version 7.6 (Applied Maths). Results were interpreted according to the criteria as previously reported (Tenover et al. 1995).

For wgSNP analysis, DNA was extracted using the DNeasy UltraClean Microbial Kit (Qiagen). WGS was performed using Miseq system with Miseq Reagent kit v3 (600 cycles) (Illumina) (Mitsumoto‐Kaseida et al. 2025). The reads were trimmed and assembled using CLC Genomics Workbench (Qiagen). WgSNP analyses for *K. pneumoniae* and the *E. cloacae* complex were conducted at different time points; therefore, different versions of CLC Genomics Workbench were used (v24.0 for *K. pneumoniae* and v22.1.2 for the *E. cloacae* complex). Within each species, all isolates were analyzed using the same software version. An SNP matrix was constructed using CLC Microbial Genomics Module 24.0 for *K. pneumoniae* or 22.1.2 for the *E. cloacae* complex by detecting SNPs against the reference genomes of *K. pneumoniae* (accession number: NZ_CP128487) or *E. cloacae* (accession number: NZ_CP014280), and pairwise SNP comparison was performed. There is no clear threshold of SNP numbers to determine the relatedness between isolates (Miro et al. 2019; Schürch et al. 2018). However, a previous study on *K. pneumoniae* reported that core‐genome (cg) SNP analysis showed 6–29 SNPs in isolates with identical cgMLST profiles and 16–61 SNPs among ST405 isolates (Miro et al. 2019). Additionally, WGS analysis detected differences in the internal strains between clades ranging from 9 to 131 SNPs (Jiang et al. 2015). Therefore, SNP thresholds for genetic relatedness were determined with reference to ranges reported in those previous studies (Miro et al. 2019; Jiang et al. 2015) and based on the SNP distributions observed in this study.

### 2.4 Discriminatory Power of and Concordance Between the Methods

The discriminatory power of the methods and their concordance were assessed using Simpson's index of diversity (SID) and adjusted Rand index (aRI), respectively. The data were analyzed using the online tool for quantitative assessment of classification agreement (www.comparingpartitions.info) (Hu et al. 2021; Jun et al. 2023; Hu et al. 2023).

## 3 Results

Of the 66 isolates (20 ESBL‐producing *K. pneumoniae*, 15 IMP‐producing *K. pneumoniae*, and 31 IMP‐producing *E. cloacae* complex isolates) that were analyzed, only two ESBL‐producing *K. pneumoniae* isolates showed discordant results between the first and second assays using the IR Biotyper, requiring a third assay to determine the confirmed results.

The pairwise SNP differences analyzed using WGS are presented in Supplementary Tables 1–3. The number of SNPs between isolates ranged from 1 to 112,686 among 231 pairs for 20 ESBL‐producing *K. pneumoniae* and 2 reference strains (≤ 6 SNPs in 4 pairs and ≥ 1892 SNPs in 227 pairs). For 15 IMP‐producing *K. pneumoniae* and 2 reference strains, the range was 1–124,950 SNPs among 136 pairs (≤ 16 SNPs in 22 pairs and ≥ 10,662 SNPs in 114 pairs). In the case of 31 IMP‐producing *E. cloacae* complex isolates, the SNP differences ranged from 0 to 106,232 SNPs among 465 pairs (≤ 33 SNPs in 120 pairs, between 89 and 94 SNPs in 4 pairs, and ≥ 10715 SNPs in 341 pairs). In this study, the thresholds of relatedness were considered to be ≤ 16 SNPs for *K. pneumoniae* and ≤ 94 SNPs for the *E. cloacae* complex.

**Figure 1:**
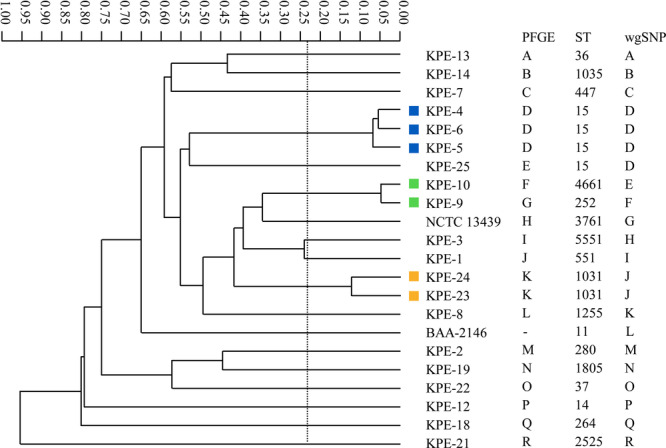
Dendrogram of ESBL‐producing *K. pneumoniae* obtained using the IR Biotyper and correlation between the IR Biotyper and comparator methods. The STs of some isolates have been reported in our previous study (Higashino et al. 2017). The same colors and alphabets indicate strains that were classified into clusters by the IR Biotyper, PFGE, and wgSNP analyses, respectively. Data from a single assay are shown as representative data.

**Figure 2:**
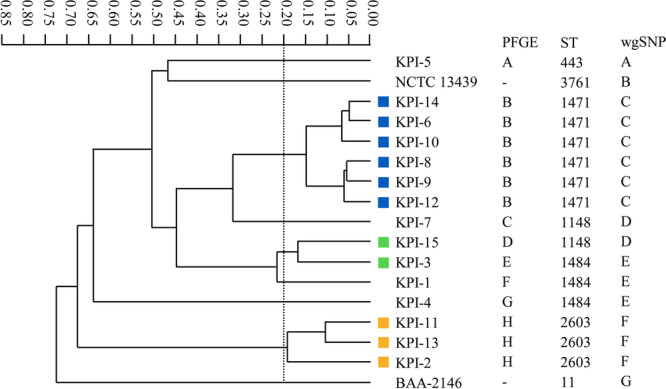
Dendrogram of IMP‐producing *K. pneumoniae* obtained using the IR Biotyper and correlation between the IR Biotyper and comparator methods. All STs and PFGE results have been reported in our previous study (Yamakawa et al. 2019). The same colors and alphabets indicate strains that were classified into clusters by the IR Biotyper, PFGE, and wgSNP analyses, respectively. Data from a single assay are shown as representative data.

**Figure 3:**
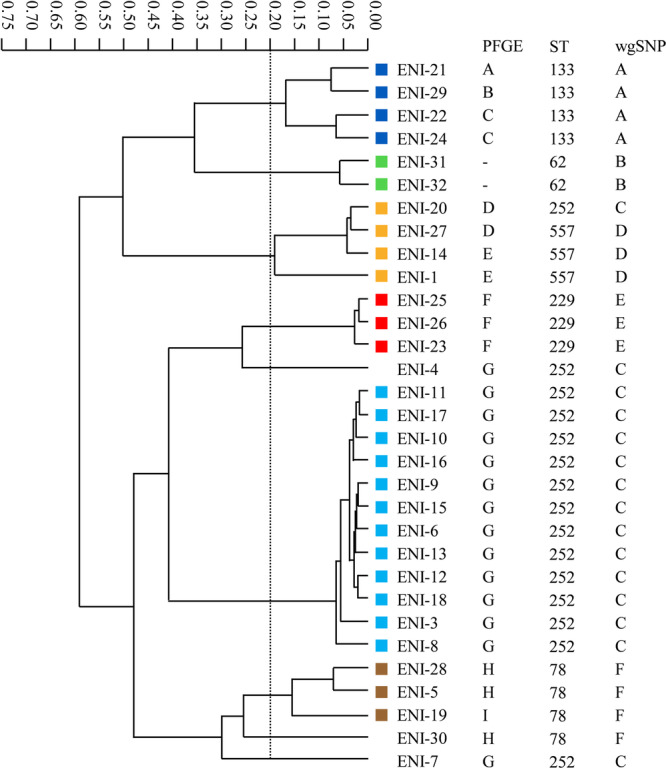
Dendrogram of IMP‐producing *E. cloacae* complex obtained using the IR Biotyper and correlation between the IR Biotyper and comparator methods. The same colors and alphabets indicate strains that were classified into clusters by the IR Biotyper, PFGE, and wgSNP analyses, respectively. Data from a single assay are shown as representative data.

Figures 1, 2, 3 show the dendrogram generated by the IR Biotyper, and the relationships between the results of the IR Biotyper and those of the comparator methods.

The IR Biotyper detected three clusters of ESBL‐producing *K. pneumoniae* isolates (Figure 1). Of these clusters, two were determined as respective clusters by PFGE and wgSNP analyses, and the strains in each of these two clusters showed the same STs. Specifically, the IR Biotyper grouped KPE‐4, KPE‐5, and KPE‐6 into a cluster, and they were determined as ST15 by MLST and as similar strains by PFGE and wgSNP analyses. Similarly, KPE‐23 and KPE‐24 were grouped into a cluster by the IR Biotyper, identified as ST1031 by MLST, and classified into the same cluster by PFGE and wgSNP analyses. Conversely, although the IR Biotyper classified KPE‐9 and KPE‐10 into a cluster, they were not considered to be similar by PFGE, MLST, and wgSNP analyses.

For the 15 IMP‐producing *K. pneumoniae* isolates, the IR Biotyper detected three clusters (Figure 2). Of these clusters, isolates in two clusters showed high concordance with the PFGE, MLST, and wgSNP analyses. Specifically, six isolates (KPI‐6, KPI‐8, KPI‐9, KPI‐10, KPI‐12, and KPI‐14) were identified as ST1471 and three isolates (KPI‐2, KPI‐11, and KPI‐13) were identified as ST2603, and were classified into respective clusters by PFGE and wgSNP analyses. Meanwhile, two isolates (KPI‐3 and KPI‐15) were classified into the same cluster by the IR Biotyper, but did not show similarities in the PFGE, MLST, and wgSNP analyses.

The IR Biotyper identified six clusters of the IMP‐producing *E. cloacae* complex isolates (Figure 3). One cluster included 12 isolates (ENI‐3, ENI‐6, ENI‐8, ENI‐9, ENI‐10, ENI‐11, ENI‐12, ENI‐13, ENI‐15, ENI‐16, ENI‐17, and ENI‐18), which were classified into a cluster by PFGE and wgSNP analyses and identified as ST252. Similarly, three isolates (ENI‐23, ENI‐25, and ENI‐26) were classified into a cluster by the IR Biotyper, PFGE, and wgSNP analyses and determined as the same ST (ST229). Three (ENI‐5, ENI‐19, and ENI‐28) and four (ENI‐21, ENI‐22, ENI‐24, and ENI‐29) isolates, which were classified into respective clusters by the IR Biotyper and wgSNP analyses, were identified as ST78 and ST133, respectively; however, several strains in these clusters showed different PFGE patterns. Four isolates (ENI‐1, ENI‐14, ENI‐20, and ENI‐27), which were classified into a cluster by the IR Biotyper, were divided into two partially different clusters in PFGE and wgSNP analyses. Specifically, one isolate was determined as ST252 and three isolates were determined as ST557. Two isolates (ENI‐31 and ENI‐32), which were classified into a cluster by the IR Biotyper and wgSNP analyses, were identified as ST62.

Overall, of the 66 strains tested, the IR Biotyper identified phenotypic clonal similarity in 46 isolates (7 ESBL‐producing *K. pneumoniae*, 11 IMP‐producing *K. pneumoniae*, and 28 IMP‐producing *E. cloacae* complex isolates) and classified them into 12 clusters (three, three, and six clusters, respectively). Among these 12 clusters, isolates included in six respective clusters (two clusters each) showed the same STs and were classified into the same clusters by PFGE and wgSNP analyses. Discordant results between the IR Biotyper and the genotyping methods (Figures 1, 2, 3) are summarized in Table 1 for cases in which all three genotyping methods reached the same interpretation (concordant or discordant).

**Table 1:** Discordant results between the IR Biotyper and genotyping methods.

Bacterium	Isolate	Typing method			
		IR Biotyper	PFGE	MLST	wgSNP
ESBL‐producing *K. pneumoniae*	KPE‐9, 10	Grouped	Not grouped	Different STs	Not grouped
IMP‐producing *K. pneumoniae*	KPI‐3, 15	Grouped	Not grouped	Different STs	Not grouped
IMP‐producing *E. cloacae* complex	ENI‐1, 14, 20, 27	Grouped	Two groups	Two STs	Two groups
IMP‐producing *E. cloacae* complex	ENI‐5, 28, 30	Two groups	Grouped	Same ST	Grouped
IMP‐producing *E. cloacae* complex	ENI‐3, 6, 7, 8, 9, 10, 11, 12, 13, 15, 16, 17, 18	Two groups	Grouped	Same ST	Grouped

*Note:* The table lists discordant results between the IR Biotyper and the genotyping methods. Only cases in which all three genotyping methods (PFGE, MLST, and wgSNP) reached the same interpretation (concordant or discordant) are included. The composition of each group and any exceptions can be found in Figures 1, 2, 3.

The SID ranged from 0.817 to 0.978 by the IR Biotyper, 0.754 to 0.981 by PFGE, and 0.733 to 0.970 by MLST or wgSNP analyses. The ranges of aRI were as follows: 0.734–0.967 between the IR Biotyper and PFGE, 0.658–0.857 between the IR Biotyper and MLST or wgSNP analyses, 0.720–0.877 between PFGE and MLST or wgSNP analyses, and 1.000 between MLST and wgSNP analyses (Table 2).

**Table 2:** Discriminatory power of and concordance between the methods.

Bacterium	Typing method	SID (95% CI)	aRI			
			IR Biotyper	PFGE^a^	MLST	wgSNP
ESBL‐producing *K. pneumoniae*	IR Biotyper	0.978 (0.950–1.000)	—			
	PFGE^a^	0.981 (0.951–1.000)	0.866	—		
	MLST	0.970 (0.925–1.000)	0.658	0.720	—	
	wgSNP	0.970 (0.925–1.000)	0.658	0.720	1.000	—
IMP‐producing *K. pneumoniae*	IR Biotyper	0.860 (0.737–0.983)	—			
	PFGE^a^	0.829 (0.674–0.983)	0.967	—		
	MLST	0.838 (0.729–0.947)	0.857	0.877	—	
	wgSNP	0.838 (0.729–0.947)	0.857	0.877	1.000	—
IMP‐producing *E. cloacae* complex	IR Biotyper	0.817 (0.714–0.921)	—			
	PFGE^a^	0.754 (0.602–0.905)	0.734	—		
	MLST	0.733 (0.598–0.868)	0.725	0.846	—	
	wgSNP	0.733 (0.598–0.868)	0.725	0.846	1.000	—

Abbreviations: aRI, adjusted Rand index; CI, confidence interval; SID, Simpson's index of diversity.

^a^ The strains not determined by PFGE were excluded from the calculation of the SID for PFGE and aRI between PFGE and other analyses.

## 4 Discussion

Our study demonstrated the performance of the IR Biotyper for the detection of bacterial phenotypic similarities in ESBL‐ and IMP‐producing Enterobacterales isolated in our hospital. Using this system, we detected 12 clusters from the 66 isolates tested in this study and a correlation between the results of IR Biotyper and those of genotyping methods, such as PFGE, MLST, and wgSNP analyses. Recent studies have reported that the IR Biotyper is a promising tool for strain typing of Enterobacterales and other Gram‐negative bacteria (Martak et al. 2019; Hu et al. 2021; Vogt et al. 2019; Rakovitsky et al. 2020). For *K. pneumoniae*, it was previously reported that the aRI between the IR biotyper and PFGE or SNP‐based core‐genome analysis was 0.743, but the aRI between the IR Biotyper and MLST was 0.192–1.000. For the *E. cloacae* complex, previous studies reported that the aRI between the IR biotyper and MLST or SNP‐based clustering was 0.801–0.963 or 0.436–0.818 (Martak et al. 2019; Vogt et al. 2019; Wendel et al. 2022).

There are several carbapenemases, including *K. pneumoniae* carbapenemases (KPCs), OXA‐β‐lactamases, and MBLs (New Delhi MBL [NDM], Verona integron‐encoded MBL [VIM], and IMP‐type MBL). A previous study reported an outbreak of KPC‐3‐producing *K. pneumoniae*, which was analyzed using FT‐IR spectroscopy (Silva et al. 2020). In contrast to Europe and the United States, IMP‐type is the most prevalent in Japan. We previously reported the possibility that IMP‐1‐producing *K. pneumoniae* was transmitted among patients in our hospital (Yamakawa et al. 2019). The molecular and epidemiological characteristics of drug‐resistant bacteria differ geographically. We performed this study because these differences might affect assay results. Our results support the versatility of FT‐IR spectroscopy in typing carbapenemase‐producing Enterobacterales in clinical settings, based on analysis of IMP‐producing *K. pneumoniae* and *E. cloacae* complex isolates from the university hospital in western Japan.

Regarding the reproducibility of this assay, 64 of the 66 tested isolates showed concordant results between the first and second assays, and the results of the remaining two isolates were confirmed in the third assay. Technically, culture conditions, such as agar medium used, incubation time, and temperature, must be standardized to effectively utilize the discriminatory performance of the IR Biotyper, and these conditions depend on the organism.

Additionally, genotyping methods are time‐consuming and labor‐intensive, whereas assays using the IR Biotyper can be performed rapidly and easily. Therefore, the IR Biotyper might improve the early detection of ESBL‐ and IMP‐producing Enterobacterales outbreaks if assays are performed in a timely manner. Meanwhile, we observed some discordant results between the IR Biotyper and genotyping methods (Table 1), indicating that the resolution of FT‐IR–based clustering may be insufficient to distinguish certain strains that are grouped into same clusters by genotyping. Therefore, clusters identified by the IR Biotyper should be interpreted as preliminary findings, and confirmatory genotyping remains necessary for definitive outbreak investigation.

This study had some limitations. First, we conducted this study using stored isolates and did not assess the IR Biotyper in real time during the outbreak. However, a previous study has shown that FT‐IR spectroscopy could be a valuable tool for rapid outbreak analysis (Vogt et al. 2019). Second, reference strains for the *E. cloacae* complex were not included in this study because they were not available in our collection, which may limit the external validation of the clustering results. Nevertheless, the inclusion of multiple genotyping comparator methods provided robust internal validation of the clustering results within the study setting. Third, because this study focused on ESBL‐ and IMP‐producing Enterobacterales, we did not evaluate the usefulness of the IR Biotyper against other bacteria. However, a recent study reported that FT‐IR spectroscopy quickly detected clusters of *Pseudomonas aeruginosa* and *Acinetobacter baumannii* in addition to Enterobacterales (Martak et al. 2019). Finally, we used SNP thresholds determined with reference to previous studies and based on SNP distributions observed in this study. Therefore, the use of alternative thresholds could influence isolate classification.

In conclusion, we revealed the performance of the IR Biotyper for the detection of clonal similarity among ESBL‐ and IMP‐producing Enterobacterales and it might be a useful tool for investigating outbreaks because of its simple and rapid nature.

## Author Contributions

**Yasuhide Kawamoto:** methodology; investigation. **Kosuke Kosai:** conceptualization; writing – original draft; writing – review and editing; methodology; funding acquisition. **Mika Murata:** methodology; investigation. **Kenji Ota:** writing – review and editing. **Fujiko Mitsumoto‐Kaseida:** writing – review and editing. **Norihito Kaku:** writing – review and editing. **Hiroo Hasegawa:** writing – review and editing. **Koichi Izumikawa:** writing – review and editing. **Hiroshi Mukae:** writing – review and editing. **Katsunori Yanagihara:** writing – review and editing; funding acquisition.

## Ethics Statement

This study was approved by the Institutional Review Board of Nagasaki University Hospital (approval number: 20091424).

## Conflicts of Interest

Reagents and instrumentation were provided by Bruker Japan K.K. Kosuke Kosai and Katsunori Yanagihara received honoraria from Bruker Japan K.K.

## Disclosure of Generative AI Use in Writing

We used “Paperpal” to improve the readability of the manuscript. We have also had the paper reviewed and edited and take full responsibility for its content.

## Supporting information

supplementary table.

## Data Availability

Data will be made available on request to the authors.
