# Supplementary material for: Rapid Typing of Extended‐Spectrum β‐Lactamase (ESBL)‐ and Metallo‐β‐Lactamase (MBL)‐Producing Enterobacterales Using Fourier Transform Infrared (FT‐IR) Spectroscopy
Source: Microbiologyopen. 2026 Feb 2;15(1):e70218. doi: 10.1002/mbo3.70218 (PMC12863417; doi:10.1002/mbo3.70218)
Supplement: Supplementary file 1 — supplementary table. [file MBO3-15-e70218-s001.docx]

Supplementary Table 1 Difference matrix for the number of SNPs analyzed using WGS for ESBL-producing *Klebsiella pneumoniae*

|  | KPE-1 | KPE-2 | KPE-3 | KPE-4 | KPE-5 | KPE-6 | KPE-7 | KPE-8 | KPE-9 | KPE-10 | KPE-12 | KPE-13 | KPE-14 | KPE-18 | KPE-19 | KPE-21 | KPE-22 | KPE-23 | KPE-24 | KPE-25 | NCTC 13439 | BAA-2146 |
| --- | --- | --- | --- | --- | --- | --- | --- | --- | --- | --- | --- | --- | --- | --- | --- | --- | --- | --- | --- | --- | --- | --- |
| KPE-1 | 0 |  |  |  |  |  |  |  |  |  |  |  |  |  |  |  |  |  |  |  |  |  |
| KPE-2 | 9703 | 0 |  |  |  |  |  |  |  |  |  |  |  |  |  |  |  |  |  |  |  |  |
| KPE-3 | 106705 | 106823 | 0 |  |  |  |  |  |  |  |  |  |  |  |  |  |  |  |  |  |  |  |
| KPE-4 | 7538 | 6723 | 105436 | 0 |  |  |  |  |  |  |  |  |  |  |  |  |  |  |  |  |  |  |
| KPE-5 | 7542 | 6727 | 105442 | 6 | 0 |  |  |  |  |  |  |  |  |  |  |  |  |  |  |  |  |  |
| KPE-6 | 7538 | 6723 | 105438 | 2 | 6 | 0 |  |  |  |  |  |  |  |  |  |  |  |  |  |  |  |  |
| KPE-7 | 94688 | 94803 | 112486 | 93398 | 93404 | 93400 | 0 |  |  |  |  |  |  |  |  |  |  |  |  |  |  |  |
| KPE-8 | 94786 | 94878 | 112661 | 93554 | 93560 | 93556 | 14275 | 0 |  |  |  |  |  |  |  |  |  |  |  |  |  |  |
| KPE-9 | 10498 | 9753 | 106893 | 7555 | 7559 | 7555 | 95125 | 95227 | 0 |  |  |  |  |  |  |  |  |  |  |  |  |  |
| KPE-10 | 9719 | 8065 | 106821 | 6933 | 6937 | 6933 | 94779 | 94944 | 9695 | 0 |  |  |  |  |  |  |  |  |  |  |  |  |
| KPE-12 | 8390 | 7721 | 106029 | 2862 | 2866 | 2862 | 94065 | 94193 | 8558 | 7882 | 0 |  |  |  |  |  |  |  |  |  |  |  |
| KPE-13 | 10534 | 9379 | 107165 | 7521 | 7525 | 7521 | 95024 | 95097 | 10228 | 9430 | 8498 | 0 |  |  |  |  |  |  |  |  |  |  |
| KPE-14 | 10212 | 9587 | 106866 | 7539 | 7543 | 7539 | 94918 | 94969 | 10315 | 9779 | 8370 | 10299 | 0 |  |  |  |  |  |  |  |  |  |
| KPE-18 | 95179 | 95135 | 112686 | 93765 | 93771 | 93767 | 15878 | 15604 | 95485 | 95181 | 94463 | 95364 | 95279 | 0 |  |  |  |  |  |  |  |  |
| KPE-19 | 10148 | 9222 | 106802 | 7337 | 7341 | 7337 | 94658 | 94723 | 9987 | 9260 | 8390 | 9802 | 10189 | 95003 | 0 |  |  |  |  |  |  |  |
| KPE-21 | 10495 | 9554 | 107209 | 7555 | 7559 | 7555 | 94845 | 94914 | 10236 | 9474 | 8577 | 9821 | 10350 | 95288 | 9723 | 0 |  |  |  |  |  |  |
| KPE-22 | 10501 | 9707 | 107190 | 7740 | 7744 | 7740 | 94894 | 95021 | 10265 | 9637 | 8700 | 10023 | 10383 | 95302 | 9509 | 9849 | 0 |  |  |  |  |  |
| KPE-23 | 106331 | 106344 | 12574 | 104917 | 104923 | 104919 | 112279 | 112380 | 106367 | 106291 | 105599 | 106683 | 106382 | 112437 | 106307 | 106722 | 106706 | 0 |  |  |  |  |
| KPE-24 | 106332 | 106345 | 12575 | 104918 | 104924 | 104920 | 112279 | 112381 | 106368 | 106292 | 105600 | 106684 | 106383 | 112437 | 106308 | 106723 | 106707 | 1 | 0 |  |  |  |
| KPE-25 | 7318 | 6308 | 105025 | 1892 | 1896 | 1892 | 92990 | 93137 | 7214 | 6531 | 3737 | 7121 | 7216 | 93365 | 6947 | 7181 | 7345 | 104481 | 104482 | 0 |  |  |
| NCTC 13439 | 10507 | 9525 | 107025 | 7561 | 7565 | 7561 | 94771 | 94891 | 10296 | 9273 | 8566 | 9852 | 10426 | 95099 | 9437 | 9978 | 9609 | 106509 | 106510 | 7149 | 0 |  |
| BAA-2146 | 11469 | 10508 | 107979 | 8442 | 8446 | 8442 | 95862 | 95992 | 11092 | 10539 | 9450 | 10293 | 11117 | 96155 | 10562 | 10780 | 9289 | 107379 | 107380 | 8149 | 10845 | 0 |

Supplementary Table 2 Difference matrix for the number of SNPs analyzed using WGS for IMP-producing *Klebsiella pneumoniae*

|  | KPI-1 | KPI-2 | KPI-3 | KPI-4 | KPI-5 | KPI-6 | KPI-7 | KPI-8 | KPI-9 | KPI-10 | KPI-11 | KPI-12 | KPI-13 | KPI-14 | KPI-15 | NCTC 13439 | BAA-2146 |
| --- | --- | --- | --- | --- | --- | --- | --- | --- | --- | --- | --- | --- | --- | --- | --- | --- | --- |
| KPI-1 | 0 |  |  |  |  |  |  |  |  |  |  |  |  |  |  |  |  |
| KPI-2 | 120318 | 0 |  |  |  |  |  |  |  |  |  |  |  |  |  |  |  |
| KPI-3 | 2 | 120318 | 0 |  |  |  |  |  |  |  |  |  |  |  |  |  |  |
| KPI-4 | 16 | 120311 | 16 | 0 |  |  |  |  |  |  |  |  |  |  |  |  |  |
| KPI-5 | 120367 | 13809 | 120367 | 120358 | 0 |  |  |  |  |  |  |  |  |  |  |  |  |
| KPI-6 | 120734 | 14595 | 120734 | 120725 | 14319 | 0 |  |  |  |  |  |  |  |  |  |  |  |
| KPI-7 | 124950 | 106949 | 124950 | 124939 | 106947 | 106799 | 0 |  |  |  |  |  |  |  |  |  |  |
| KPI-8 | 120738 | 14599 | 120738 | 120729 | 14323 | 4 | 106803 | 0 |  |  |  |  |  |  |  |  |  |
| KPI-9 | 120733 | 14595 | 120733 | 120724 | 14319 | 1 | 106798 | 5 | 0 |  |  |  |  |  |  |  |  |
| KPI-10 | 120735 | 14600 | 120735 | 120726 | 14324 | 8 | 106799 | 12 | 7 | 0 |  |  |  |  |  |  |  |
| KPI-11 | 120316 | 7 | 120316 | 120309 | 13808 | 14594 | 106948 | 14598 | 14594 | 14599 | 0 |  |  |  |  |  |  |
| KPI-12 | 120733 | 14595 | 120733 | 120724 | 14319 | 3 | 106798 | 7 | 2 | 7 | 14594 | 0 |  |  |  |  |  |
| KPI-13 | 120318 | 8 | 120318 | 120311 | 13809 | 14595 | 106949 | 14599 | 14595 | 14600 | 5 | 14595 | 0 |  |  |  |  |
| KPI-14 | 120733 | 14595 | 120733 | 120724 | 14319 | 3 | 106798 | 7 | 2 | 9 | 14594 | 4 | 14595 | 0 |  |  |  |
| KPI-15 | 124947 | 106946 | 124947 | 124936 | 106943 | 106795 | 7 | 106799 | 106794 | 106795 | 106945 | 106794 | 106946 | 106794 | 0 |  |  |
| NCTC 13439 | 120202 | 13993 | 120202 | 120199 | 14015 | 14691 | 106600 | 14695 | 14692 | 14697 | 13992 | 14692 | 13993 | 14692 | 106596 | 0 |  |
| BAA-2146 | 121128 | 10664 | 121128 | 121119 | 14837 | 15268 | 107896 | 15272 | 15268 | 15275 | 10662 | 15270 | 10664 | 15268 | 107892 | 14958 | 0 |

Supplementary Table 3 Difference matrix for the number of SNPs analyzed using WGS for IMP-producing *Enterobacter cloacae* complex

|  | ENI-1 | ENI-3 | ENI-4 | ENI-5 | ENI-6 | ENI-7 | ENI-8 | ENI-9 | ENI-10 | ENI-11 | ENI-12 | ENI-13 | ENI-14 | ENI-15 | ENI-16 | ENI-17 | ENI-18 | ENI-19 | ENI-20 | ENI-21 | ENI-22 | ENI-23 | ENI-24 | ENI-25 | ENI-26 | ENI-27 | ENI-28 | ENI-29 | ENI-30 | ENI-31 | ENI-32 |
| --- | --- | --- | --- | --- | --- | --- | --- | --- | --- | --- | --- | --- | --- | --- | --- | --- | --- | --- | --- | --- | --- | --- | --- | --- | --- | --- | --- | --- | --- | --- | --- |
| ENI-1 | 0 |  |  |  |  |  |  |  |  |  |  |  |  |  |  |  |  |  |  |  |  |  |  |  |  |  |  |  |  |  |  |
| ENI-3 | 100965 | 0 |  |  |  |  |  |  |  |  |  |  |  |  |  |  |  |  |  |  |  |  |  |  |  |  |  |  |  |  |  |
| ENI-4 | 100965 | 7 | 0 |  |  |  |  |  |  |  |  |  |  |  |  |  |  |  |  |  |  |  |  |  |  |  |  |  |  |  |  |
| ENI-5 | 39993 | 92506 | 92506 | 0 |  |  |  |  |  |  |  |  |  |  |  |  |  |  |  |  |  |  |  |  |  |  |  |  |  |  |  |
| ENI-6 | 100962 | 18 | 19 | 92503 | 0 |  |  |  |  |  |  |  |  |  |  |  |  |  |  |  |  |  |  |  |  |  |  |  |  |  |  |
| ENI-7 | 100947 | 31 | 28 | 92482 | 27 | 0 |  |  |  |  |  |  |  |  |  |  |  |  |  |  |  |  |  |  |  |  |  |  |  |  |  |
| ENI-8 | 100960 | 17 | 18 | 92501 | 13 | 26 | 0 |  |  |  |  |  |  |  |  |  |  |  |  |  |  |  |  |  |  |  |  |  |  |  |  |
| ENI-9 | 100963 | 16 | 17 | 92504 | 16 | 29 | 15 | 0 |  |  |  |  |  |  |  |  |  |  |  |  |  |  |  |  |  |  |  |  |  |  |  |
| ENI-10 | 100964 | 4 | 5 | 92505 | 16 | 29 | 15 | 14 | 0 |  |  |  |  |  |  |  |  |  |  |  |  |  |  |  |  |  |  |  |  |  |  |
| ENI-11 | 100959 | 15 | 16 | 92500 | 11 | 20 | 10 | 13 | 13 | 0 |  |  |  |  |  |  |  |  |  |  |  |  |  |  |  |  |  |  |  |  |  |
| ENI-12 | 100963 | 7 | 4 | 92506 | 19 | 28 | 18 | 17 | 5 | 16 | 0 |  |  |  |  |  |  |  |  |  |  |  |  |  |  |  |  |  |  |  |  |
| ENI-13 | 100959 | 17 | 14 | 92500 | 13 | 18 | 12 | 15 | 15 | 6 | 14 | 0 |  |  |  |  |  |  |  |  |  |  |  |  |  |  |  |  |  |  |  |
| ENI-14 | 15 | 100968 | 100968 | 39996 | 100965 | 100950 | 100961 | 100966 | 100967 | 100962 | 100966 | 100962 | 0 |  |  |  |  |  |  |  |  |  |  |  |  |  |  |  |  |  |  |
| ENI-15 | 100967 | 20 | 21 | 92508 | 20 | 33 | 19 | 4 | 18 | 17 | 21 | 19 | 100970 | 0 |  |  |  |  |  |  |  |  |  |  |  |  |  |  |  |  |  |
| ENI-16 | 100958 | 15 | 16 | 92500 | 11 | 20 | 10 | 13 | 13 | 4 | 16 | 6 | 100961 | 17 | 0 |  |  |  |  |  |  |  |  |  |  |  |  |  |  |  |  |
| ENI-17 | 100956 | 13 | 14 | 92497 | 9 | 22 | 4 | 11 | 11 | 6 | 14 | 8 | 100959 | 15 | 6 | 0 |  |  |  |  |  |  |  |  |  |  |  |  |  |  |  |
| ENI-18 | 100959 | 17 | 14 | 92500 | 13 | 18 | 12 | 15 | 15 | 6 | 14 | 4 | 100962 | 19 | 6 | 8 | 0 |  |  |  |  |  |  |  |  |  |  |  |  |  |  |
| ENI-19 | 39991 | 92507 | 92507 | 2 | 92504 | 92483 | 92502 | 92505 | 92506 | 92501 | 92507 | 92501 | 39994 | 92509 | 92501 | 92498 | 92501 | 0 |  |  |  |  |  |  |  |  |  |  |  |  |  |
| ENI-20 | 100956 | 13 | 14 | 92497 | 9 | 22 | 4 | 11 | 11 | 6 | 14 | 8 | 100959 | 15 | 6 | 0 | 8 | 92498 | 0 |  |  |  |  |  |  |  |  |  |  |  |  |
| ENI-21 | 29333 | 100103 | 100104 | 39215 | 100100 | 100084 | 100096 | 100101 | 100102 | 100097 | 100104 | 100098 | 29334 | 100105 | 100097 | 100094 | 100098 | 39213 | 100094 | 0 |  |  |  |  |  |  |  |  |  |  |  |
| ENI-22 | 29305 | 100077 | 100078 | 39183 | 100074 | 100058 | 100071 | 100075 | 100076 | 100071 | 100078 | 100072 | 29307 | 100079 | 100071 | 100068 | 100072 | 39181 | 100068 | 94 | 0 |  |  |  |  |  |  |  |  |  |  |
| ENI-23 | 106229 | 30467 | 30468 | 99406 | 30465 | 30463 | 30464 | 30467 | 30467 | 30462 | 30468 | 30462 | 106232 | 30470 | 30462 | 30460 | 30462 | 99407 | 30460 | 105398 | 105367 | 0 |  |  |  |  |  |  |  |  |  |
| ENI-24 | 29304 | 100075 | 100076 | 39182 | 100072 | 100056 | 100069 | 100073 | 100074 | 100069 | 100076 | 100070 | 29306 | 100077 | 100069 | 100066 | 100070 | 39180 | 100066 | 92 | 4 | 105365 | 0 |  |  |  |  |  |  |  |  |
| ENI-25 | 106229 | 30467 | 30468 | 99405 | 30465 | 30463 | 30464 | 30467 | 30467 | 30462 | 30468 | 30462 | 106232 | 30470 | 30462 | 30460 | 30462 | 99406 | 30460 | 105398 | 105367 | 2 | 105365 | 0 |  |  |  |  |  |  |  |
| ENI-26 | 106228 | 30466 | 30467 | 99405 | 30464 | 30462 | 30463 | 30466 | 30466 | 30461 | 30467 | 30461 | 106231 | 30469 | 30461 | 30459 | 30461 | 99406 | 30459 | 105397 | 105366 | 1 | 105364 | 1 | 0 |  |  |  |  |  |  |
| ENI-27 | 9 | 100968 | 100968 | 39996 | 100965 | 100950 | 100963 | 100966 | 100967 | 100962 | 100966 | 100962 | 18 | 100970 | 100961 | 100959 | 100962 | 39994 | 100959 | 29336 | 29308 | 106232 | 29307 | 106232 | 106231 | 0 |  |  |  |  |  |
| ENI-28 | 39994 | 92507 | 92507 | 1 | 92504 | 92483 | 92502 | 92505 | 92506 | 92501 | 92507 | 92501 | 39997 | 92509 | 92501 | 92498 | 92501 | 3 | 92498 | 39216 | 39184 | 99407 | 39183 | 99406 | 99406 | 39997 | 0 |  |  |  |  |
| ENI-29 | 29330 | 100100 | 100101 | 39212 | 100097 | 100081 | 100093 | 100098 | 100099 | 100094 | 100101 | 100095 | 29331 | 100102 | 100094 | 100091 | 100095 | 39210 | 100091 | 3 | 91 | 105395 | 89 | 105395 | 105394 | 29333 | 39213 | 0 |  |  |  |
| ENI-30 | 39995 | 92508 | 92508 | 2 | 92505 | 92484 | 92503 | 92506 | 92507 | 92502 | 92508 | 92502 | 39998 | 92510 | 92502 | 92499 | 92502 | 4 | 92499 | 39217 | 39185 | 99408 | 39184 | 99407 | 99407 | 39998 | 1 | 39214 | 0 |  |  |
| ENI-31 | 29184 | 100214 | 100215 | 39080 | 100211 | 100195 | 100209 | 100212 | 100213 | 100208 | 100215 | 100209 | 29187 | 100216 | 100208 | 100205 | 100209 | 39078 | 100205 | 10745 | 10717 | 105485 | 10715 | 105485 | 105484 | 29187 | 39081 | 10742 | 39082 | 0 |  |
| ENI-32 | 29184 | 100214 | 100215 | 39080 | 100211 | 100195 | 100209 | 100212 | 100213 | 100208 | 100215 | 100209 | 29187 | 100216 | 100208 | 100205 | 100209 | 39078 | 100205 | 10745 | 10717 | 105485 | 10715 | 105485 | 105484 | 29187 | 39081 | 10742 | 39082 | 0 | 0 |
